# Emergency department visits, ambulance calls, and mortality associated with an exceptional heat wave in Sydney, Australia, 2011: a time-series analysis

**DOI:** 10.1186/1476-069X-11-3

**Published:** 2012-01-24

**Authors:** Andrea Schaffer, David Muscatello, Richard Broome, Stephen Corbett, Wayne Smith

**Affiliations:** 1 Centre for Epidemiology and Research, New South Wales Department of Health, Sydney, Australia; 2 Environmental Health Branch, New South Wales Department of Health, Sydney, Australia; 3 Centre for Population Health, Sydney West Area Health Service, Sydney, Australia

**Keywords:** Heat wave, Australia, Emergency department, Ambulance, Mortality, Syndromic surveillance

## Abstract

**Background:**

From January 30-February 6, 2011, New South Wales was affected by an exceptional heat wave, which broke numerous records. Near real-time Emergency Department (ED) and ambulance surveillance allowed rapid detection of an increase in the number of heat-related ED visits and ambulance calls during this period. The purpose of this study was to quantify the excess heat-related and all-cause ED visits and ambulance calls, and excess all-cause mortality, associated with the heat wave.

**Methods:**

ED and ambulance data were obtained from surveillance and administrative databases, while mortality data were obtained from the state death registry. The observed counts were compared with the average counts from the same period from 2006/07 through 2009/10, and a Poisson regression model was constructed to calculate the number of excess ED visits, ambulance and deaths after adjusting for calendar and lag effects.

**Results:**

During the heat wave there were 104 and 236 ED visits for heat effects and dehydration respectively, and 116 ambulance calls for heat exposure. From the regression model, all-cause ED visits increased by 2% (95% CI 1.01-1.03), all-cause ambulance calls increased by 14% (95% CI 1.11-1.16), and all-cause mortality increased by 13% (95% CI 1.06-1.22). Those aged 75 years and older had the highest excess rates of all outcomes.

**Conclusions:**

The 2011 heat wave resulted in an increase in the number of ED visits and ambulance calls, especially in older persons, as well as an increase in all-cause mortality. Rapid surveillance systems provide markers of heat wave impacts that have fatal outcomes.

## Background

Heat wave events can have considerable adverse impacts on morbidity and mortality. During an extreme heat wave in France in 2003, maximum daily temperatures exceeded 35°C for 15 days; the number of excess deaths across the country due to the heat wave was estimated to be 14,800, equivalent to a 60% increase over the expected count [1]. In Paris alone, there were 2228 excess deaths with a 5-fold increased risk of death observed on the hottest day of the heat wave [2].

In Chicago in 1995 there was a 5-day heat wave which peaked on July 13 with a maximum temperature of 40°C [3]. During the month of July there were an estimated 696 excess deaths, equivalent to a 30% increase. As is often observed, the effect of the heat wave was delayed, with the greatest daily increase in mortality recorded two days after the hottest heat wave day, with an excess of 275 deaths [4]. Smaller increases in mortality during less severe heat waves have also been observed in Europe [5-9], China [10], and the United States [11,12].

It has been suggested that some of the excess deaths observed during heat waves are due to "mortality displacement", meaning that the deaths of patients who would have died in the coming days or weeks are shifted forward. Evidence from France and Chicago in 2003 and 1995 suggest that mortality displacement accounts for only a small percentage of the excess deaths [2,13], but results from other studies have been inconsistent [14].

Hospitalisations have also been found to increase during extreme heat events, especially among older persons [7,15-17], although this finding is not universal [18]. The effect of heat waves on emergency department visits and ambulance calls is less well established, but the existing evidence suggests that they also increase [17,19-21]. During an 18-day heat wave in 2006 in California an excess of 16,000 ED visits was observed, including 2134 excess visits for heat-related illness [20].

The elderly are most susceptible to the effects of extreme heat [22], due to physiological changes that occur with ageing, chronic illness, certain medications, and sedentary lifestyles which contribute to reduced body temperature regulation and dehydration [23]. Other vulnerable populations include those with cardiovascular [24], respiratory [6,25], or renal disease [25,26], and those with mental health problems [27,28].

In Australia, although the more general association of increasing temperature and mortality has been studied [29], there are few studies that focus on the outcomes of specific heat wave events. A study from Adelaide of two extreme heat waves in 2008 and 2009 found an increase in ambulance calls of 10% and 16% respectively, and an increase in ED visits of 6% only during the 2008 heat wave. There was no increase in mortality for all ages in either heat wave [17], while a study from Brisbane of a 20-day heat wave found an excess of 75 deaths [30].

New South Wales (NSW) is the most populous state in Australia with a population of over 7 million people [31], the majority of whom live near the coast, which has a temperate climate. From January 30 to February 6, 2011, there was an exceptional heat wave in NSW. In the Hunter Valley, there was a record-breaking consecutive 6 days with a maximum temperature above 39°C. Overnight temperatures were similarly extreme, with Sydney experiencing 5 nights above 24°C, including a record-breaking overnight temperature of 27.6°C on February 6 [32].

Syndromic surveillance of emergency departments (EDs) is a useful tool for public health monitoring [33-35], including during heat wave events [36,37]. The NSW Ministry of Health operates near real-time syndromic surveillance of ED presentations [38] and ambulance dispatches using automatic capture and analysis of electronic ED and ambulance data, and provides early warning of daily and weekly increases in disease activity in the population. During the NSW heat wave, statistically higher than expected increases in ED visits and ambulance calls were identified, both overall and for heat-related syndromes, which included heat stroke and dehydration. Thus, the purpose of the study was to quantify the number of excess ED visits and ambulance calls attributable to a period of exceptional heat in a temperate region of Australia, and to determine whether there was a concomitant increase in the number of deaths.

## Methods

### Heat wave definition

The period of January 30-February 6 was identified by the Bureau of Meteorology, Australia's national weather agency, as a period of exceptional heat in the Sydney and Hunter regions of NSW [32]. Using this independent heat wave definition offered an unbiased and objective way to define this heat wave prior to undertaking the analysis.

### Study area

**Figure 1:**
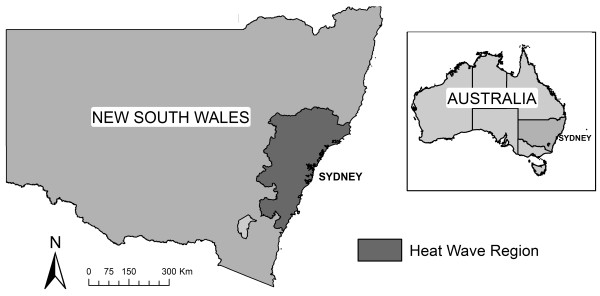
**Map of New South Wales showing heat wave region (in dark grey)**.

The study area was defined as the geographic region of NSW that experienced the maximum anomaly (6°C) above the historical average (1961-1990) during the heat wave. Geographic boundaries matching the study area were identified from the Australian Standard Geographical Classification. This region included the Statistical Divisions of Sydney, Hunter and Illawarra, and the Statistical Local Areas of Mid-Western Regional Part B, Lithgow, Oberon, Goulburn Mulwaree (Goulburn) and Goulburn Mulwaree (Bal) and comprised 8% of the land area of NSW and 73% of its population, i.e. 5.2 million people (Figure 1).

### Study period and data

The study period was restricted to the southern hemisphere summer (November 1-February 28) to reduce seasonal effects. The daily maximum and minimum overnight temperatures were obtained from the Australian Bureau of Meteorology. Data from weather stations located within the heat wave region were averaged to provide the mean maximum/minimum temperatures for the heat wave region for each day. To provide a reference period, the daily maximum and minimum temperatures for the same weather stations for the summers from 2006/07 through to 2009/10 inclusive were averaged to give the mean daily maximum and minimum temperatures over the four years.

The ED and ambulance data were obtained from a combination of an ED-based syndromic surveillance system and the Emergency Department Data Collection, which contains routinely collected data captured in the ED clinical information systems of public hospitals across NSW. The ED data contain information on the patient's demographics, as well as their reason for presenting to the ED, including the triage nurse's notes. The ambulance data is a computerised database that captures information on all ambulance dispatches in response to persons who have called emergency services, including demographics and the reason for the call. Data from the 41 EDs within the heat wave region that participate in the syndromic surveillance were available, which account for 96% of all ED visits in this region. Ambulance data for the entire study period had only been collected for the Sydney Ambulance Division region and thus the analysis of ambulance data was restricted to this area, which services approximately 82% of the population in the heat wave region. February 12 and 13, 2011, were excluded from analysis of ambulance calls as data were incomplete on those dates.

Heat-related syndromes were classified using either the International Classification of Diseases, 10th Revision (ICD-10), the International Classification of Diseases, 9th Revision (ICD-9) or the Systematized Nomenclature of Medicine (SNOMED). Heat-related ED visits included individuals assigned a provisional diagnosis of: heatstroke, sunstroke, heat syncope, heat cramp, heat exhaustion, heat fatigue, heat oedema and other effects of heat (ICD-10-AM code T67, ICD-9-AM code 992, and equivalent SNOMED codes); and exposure to excessive natural heat (ICD-10-AM code X30, ICD-9-AM code E900.0 and equivalent SNOMED codes). ED visits for dehydration included a provisional diagnosis of volume depletion (ICD-10-AM code E86, ICD-9-AM code 276.5 and equivalent SNOMED codes). The list of SNOMED codes used is available in Table S1 (see Additional file 1: Table S1). Heat-related ambulance calls were assigned a problem category of heat or cold exposure. Since only data from the summer period were included it was assumed that all of the ambulance calls assigned this problem category were for heat exposure.

All-cause mortality data were obtained from the NSW Registry of Births, Deaths, and Marriages, which records all deaths that occur in the state, and includes information on the last known residential address of the deceased. Using this information the death data were geocoded and restricted to all persons who resided within the boundaries of the heat wave region at the time of their death. Cause-specific mortality was not available at the time of the study.

This study was approved by the NSW Population and Health Services Research Ethics Committee (CINSW LNR 2011/006).

### Statistical analysis

To demonstrate the signals observed through the syndromic surveillance, a descriptive analysis was performed comparing total observed counts during the heat wave period to average observed counts from previous years for all-cause outcomes and for the more heat-specific syndromes used in the near real-time ED and ambulance surveillance system, i.e. ED visits for heat-related conditions, dehydration, and ambulance calls for heat exposure. The average observed counts were calculated by averaging the counts for each day for the summer period from 2006/07 through to 2009/10 inclusive.

Between 2006 and 2010 the population of NSW has increased by 6%, while the population over 75 years has increased by 8% and therefore ED visits, ambulance calls and deaths have been increasing over time. Thus, to control for this trend and other confounders a generalised linear model for count data, Poisson regression, was fit to determine the relative and absolute increase in the number of all-cause ED visits, all-cause ambulance calls, and all-cause deaths that were due to the heat wave. Data from the summer periods from November 1 to February 28 for the years 2006/07 through to 2010/11 inclusive were used in this analysis.

Variables for day of the week, public holidays, and quadratic variables for day of the year were included to control for calendar effects and any remaining seasonal effects and a variable for year was included to control for long term trends. An 8-day period representing the heat wave was modelled using an indicator variable set to 1 if the date fell between the start date and end date of this period, and 0 otherwise. A heat wave lag effect was considered, where the heat wave time period was shifted forward by 1, 2, 3 or 4 days. The most appropriate lag for a given outcome was determined by which model resulted in the lowest Akaike Information Criterion. As studies have found that those aged over 75 years are more susceptible to heat effects [22], for each outcome 3 analyses were performed: one for those aged < 75 years, one for those aged ≥75 years, and one for all ages. The model fit was checked using deviance and residual plots. To check for mortality displacement the period following the heat wave was modelled to check for a reduction in the number of deaths.

## Results

From January 30 through February 6, the heat wave period, the average maximum temperature in the study area was 35.2°C, and ranged from 32.0°C on January 30 to 38.5°C on February 5. The average minimum overnight temperature was 21.8°C and reached a high of 23.7°C on February 5.

**Figure 2:**
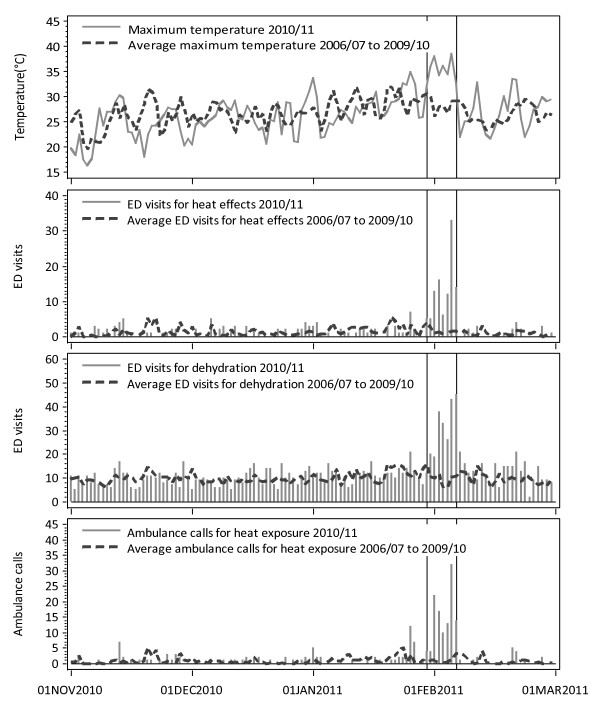
**Daily temperature and daily counts of ED visits and ambulance calls for dehydration and heat-related conditions**.

During the 8-day heat wave, there were 104 ED visits for heat effects and 236 ED visits for dehydration, and 116 ambulance calls for heat exposure (Table 1). The greatest daily counts for ED visits and ambulance calls for heat effects were observed on the hottest day of the heat wave (February 5), with 33 visits and 32 calls respectively. The highest daily count of ED visits for dehydration was observed the next day, with 45 visits. These were the highest daily counts observed in the past 5 years (Figure 2). All-cause outcomes were also higher, with 34,197 total ED visits, 8609 ambulance calls and 814 deaths during the heat wave period, as compared to an average of 30,092 visits, 7155 calls and 682 deaths in previous years, although the proportion of this increase that is due specifically to the heat wave is not clear.

**Table 1:** Observed counts of ED visits, ambulance calls and deaths during the heat wave and during the same period in previous years

	Observed (2011)	Average observed (2007-2010)
*ED visits*		
All-cause	34,197	30,092
Heat effects	104	13.5
Dehydration	236	81.3
Ambulance calls*		
All-cause	8609	7155
Heat exposure	116	8.0
*Deaths*		
All-cause	814	682

*Sydney only

From the regression modelling, the greatest relative increase in all-cause ED visits was observed with a lag of 3 days; after adjusting for calendar effects, there was a significant increase of 2% (RR 1.02, 95% CI 1.01-1.03) which corresponded to an excess of 595 (95% CI 221-973) ED visits (Table 2). Similar results were observed in those under 75 years only (RR 1.01, 95% CI 1.00-1.02), and in those over 75 years there was an increase of 8% (RR 1.08, 95% CI 1.04-1.11).

**Table 2:** Relative risk and predicted excess counts and rates based on the model with the most appropriate lag for each outcome

	Relative Risk (95% CI)	Predicted excess count (95% CI)	Predicted excess rate per 100,000 population (95% CI)
All-cause ED visits*			
All ages	1.02 (1.01-1.03)	595 (221-973)	11.5 (4.37-18.8)
< 75 years	1.01 (1.00-1.02)	336 (-9-685)	6.9 (-0.9-14.1)
≥75 years	1.08 (1.04-1.11)	322 (186-462)	100.0 (57.8-143.5)
*All-cause ambulance calls (Sydney only)^*			
All ages	1.14 (1.11-1.16)	1033 (845-1225)	24.4 (19.9-28.9)
< 75 years	1.10 (1.07-1.13)	514 (364-668)	12.9 (9.1-16.7)
≥75 years	1.17 (1.12-1.23)	323 (229-421)	135.8 (96.3-177.1)
*All-cause deaths†*			
All ages	1.13 (1.06-1.22)	96 (40-157)	1.9 (0.8-3.0)
< 75 years	1.16 (1.02-1.30)	38 (6-75)	0.8 (0.1-1.5)
≥75 years	1.12 (1.03-1.23)	58 (13-107)	18.0 (4.0-33.2)

*Lag of 3 days; ^^^Lag of 1 day; ^†^No lag

The greatest relative increase in all-cause ambulance calls was observed with a lag of 1 day; after adjustment, there was a significant increase of 14% (RR 1.14, 95% CI 1.11-1.16) corresponding to an excess of 1033 (95% CI 845-1225) calls. In the younger age group a 10% increase in the number of ambulance calls was observed (RR 1.10, 95% CI 1.07-1.13) while in those over 75 years a 17% increase was observed (RR 1.17, 95% CI 1.12-1.23).

In the adjusted model without any lag, there was a 13% increased risk of all-cause death (RR 1.13, 95% CI 1.06-1.22). This corresponded to a predicted excess of 96 deaths (95% CI 40-157). When stratified by age, in those aged less than 75 years there was a 16% increase in the number of deaths (RR 1.16, 95% CI 1.02-1.30). In persons over 75 years an increase of 12% was observed (RR 1.12, 95% CI 1.03-1.23).

## Discussion

This is one of the few studies of the impact of a heat wave event on mortality, ambulance calls and ED visits in Australia. The strength of this current study is that it combines three sources of data to form a more complete picture of the effect of a specific heat wave event, and provides useful information on the timing of the peaks of the three outcomes, which can help in predicting the effects of future heat waves.

In this study, large numbers of ED visits and ambulance calls for heat-related conditions such as heat stroke, heat exhaustion and dehydration were observed during the heat wave, with the highest counts recorded in five years, emphasizing the exceptionality of this event. The effect of the heat wave was likely exacerbated by the fact that the hottest days occurred on the weekend, when it is likely that many people would be outdoors. The largest single daily counts of ED visits and ambulance calls for heat-related conditions were recorded on the Saturday during the heat wave. The absolute count of all cause ED visits and ambulance calls were also elevated in comparison to previous years, although this increase may have been partly due to a growing population and not specific to the heat wave, hence the need for multivariate analysis to eliminate the effect of long-term trends.

The Poisson model found significant increases for all outcomes and for both age groups. As expected, those aged 75 years or older experienced the highest excess rates of ED visits, ambulance calls and deaths (Table 2). This age group also experienced the largest absolute increase in the number of deaths, with an excess of 58 deaths predicted by the model to be attributable to the heat wave period, compared to 38 in younger persons, even though they represent a smaller population. In contrast, the largest absolute increases in ED visits and ambulance calls were observed in those aged less than 75 years, which is expected as they normally account for the majority of ED visits and ambulance calls (86% and 63% respectively). Overall, the greatest absolute impact we found was on the number of ambulance calls with 1033 excess calls attributable to the heat wave, this despite the fact that the ambulance data only covered 82% of the population of the heat wave area. If ambulance data from the entire heat wave area been available, the number of excess calls would likely have been even greater.

While not as extreme as the events in France and Chicago, the impact of the heat wave on all-cause mortality overall that we observed was similar to that found in many northern hemisphere studies, despite the fact that heat effects are generally considered to be lower in regions with higher average temperatures [39], such as Australia. Although, in contrast to previous studies that have generally found a greater relative increase in mortality in older persons [7,8], in the current study the increase in mortality was greater in persons younger than 75 years. This result is similar to those of one of the few other studies of heat waves in Australia. The Nitschke et al. [17] study from Adelaide of a 2009 heat wave also found a large increase in ambulance calls, but a smaller relative increase in ED visits. They also found a large relative increase in death in younger persons (15-64 years), but in contrast they did not find any significantly increased mortality in older persons.

Unfortunately cause of death was not available at the time of the analysis and thus we were not able to investigate whether there were any cause-specific increases or to identify deaths that were potentially heat-related. But, given the wide-ranging health effects of extreme temperatures, all-cause mortality is the most sensitive measure of heat-related mortality. The period following the heat wave was modelled to check for mortality displacement. None was found, but this is difficult to assess as the effects could be obscured by subsequent hot days, as following the heat wave there were multiple days with maximum temperatures exceeding 30°C.

While the 8 days of the heat wave clearly resulted in increased health care utilisation, the extreme heat may have only been one factor in the observed results. The effect of the high heat may have been modified by air pollution and/or high humidity, both of which are associated with increased morbidity and mortality [40-42]. Both high levels of ozone and above average humidity were experienced during the heat wave period [32,43]. As well, there were certainly other periods of extreme heat during the time period of the analysis. This may underestimate the effect of this heat wave in comparison to non-heat wave periods; but, the fact that we still found significant increases in all outcomes once again suggests that this event was exceptional.

The increases in the existing heat-related syndromes included in our regular syndromic surveillance reports were plausible given the clear heat exposure. Given that delays in death registrations arising from administrative processes, the syndromic ED and ambulance data available for analysis within 24 hours may be useful as a marker of potential mortality increases that are less easily and rapidly measured. Exploration of the predictive value of these syndromes for mortality outcomes would be valuable in the design of heat wave early warning systems.

## Conclusions

This heat wave in NSW placed a great burden on the health care system. Not only was an increase in the number of heat-related ED visits and ambulance calls observed during this exceptional heat wave, but we also found a substantial impact on both all-cause ED visits and all-cause ambulance calls, especially in older populations who are known to be vulnerable to the effects of extreme heat. This occurred in tandem with an increase in the number of all-cause deaths.

## Abbreviations

ED: Emergency department; NSW: New South Wales; ICD-10-AM: International Classification of Diseases; 10th Revision; Australian Modification; ICD-9-AM: International Classification of Diseases; 9th Revision; Australian Modification; SNOMED: Systematized Nomenclature of Medicine; CI: Confidence interval.

## Competing interests

The authors declare that they have no competing interests.

## Authors' contributions

DM, RB, SC and WS conceived the study. AS contributed to the study design, conducted the analysis and drafted the manuscript. DM contributed to study design and assisted in the analysis. RB contributed to study design. All authors revised the manuscript and have read and approved the final manuscript.

## Supplementary Material

Additional file 1 **Table S1**. List of SNOMED codes used to identify ED visits for dehydration and heat-related syndromes. Click here for file

## References

[B1] Hémon D Jougla E Surmortalité liée à la canicule d'août 2003. Rapport d'étape (1-3). Estimation de la surmortalité et principales caractéristiques épidémiologiques. http://www.cepidc.inserm.fr/inserm/html/pdf/rapport_canicule_03.pdf

[B2] Le Tertre A Lefranc A Eilstein D Declercq C Medina S Blanchard M Chardon B Fabre P Filleul L Jusot JF Impact of the 2003 heatwave on all-cause mortality in 9 French cities Epidemiology 2006 17 75 79 10.1097/01.ede.0000187650.36636.1f 16357598

[B3] Centers for Disease Control and Prevention (CDC) Heat-related mortality--Chicago, July 1995 MMWR Morb Mortal Wkly Rep 1995 44 577 579 7623759

[B4] Whitman S Good G Donoghue ER Benbow N Shou W Mou S Mortality in Chicago attributed to the July 1995 heat wave Am J Public Health 1997 87 1515 1518 10.2105/AJPH.87.9.1515 9314806 PMC1380980

[B5] Rooney C McMichael AJ Kovats RS Coleman MP Excess mortality in England and Wales, and in Greater London, during the 1995 heatwave J Epidemiol Community Health 1998 52 482 486 10.1136/jech.52.8.482 9876358 PMC1756744

[B6] D'Ippoliti D Michelozzi P Marino C De'Donato F Menne B Katsouyanni K Kirchmayer U Analitis A Medina-Ramon M Paldy A The impact of heat waves on mortality in 9 European cities: results from the EuroHEAT project Environ Health 2010 9 37 45 10.1186/1476-069X-9-37 20637065 PMC2914717

[B7] Johnson H Kovats RS McGregor G Stedman J Gibbs M Walton H Cook L Black E The impact of the 2003 heat wave on mortality and hospital admissions in England Health Stat Q 2005 25 6 11 15804164

[B8] Conti S Meli P Minelli G Solimini R Toccaceli V Vichi M Beltrano C Perini L Epidemiologic study of mortality during the Summer 2003 heat wave in Italy Environ Res 2005 98 390 399 10.1016/j.envres.2004.10.009 15910795

[B9] Hertel S Le Tertre A Jockel KH Hoffmann B Quantification of the heat wave effect on cause-specific mortality in Essen, Germany Eur J Epidemiol 2009 24 407 414 10.1007/s10654-009-9359-2 19517255

[B10] Huang W Kan H Kovats S The impact of the 2003 heat wave on mortality in Shanghai, China Sci Total Environ 2010 408 2418 2420 10.1016/j.scitotenv.2010.02.009 20219235

[B11] Ostro BD Roth LA Green RS Basu R Estimating the mortality effect of the July 2006 California heat wave Environ Res 2009 109 614 619 10.1016/j.envres.2009.03.010 19394595

[B12] Anderson GB Bell ML Heat waves in the United States: mortality risk during heat waves and effect modification by heat wave characteristics in 43 U.S. communities Environ Health Perspect 2011 119 210 218 21084239 10.1289/ehp.1002313 PMC3040608

[B13] Kaiser R Le Tertre A Schwartz J Gotway CA Daley WR Rubin CH The effect of the 1995 heat wave in Chicago on all-cause and cause-specific mortality Am J Public Health 2007 97 Suppl 1 S158 S162 17413056 10.2105/AJPH.2006.100081 PMC1854989

[B14] Martiello MA Giacchi MV High temperatures and health outcomes: a review of the literature Scand J Public Health 2010 38 826 837 10.1177/1403494810377685 20688791

[B15] Mastrangelo G Fedeli U Visentin C Milan G Fadda E Spolaore P Pattern and determinants of hospitalization during heat waves: an ecologic study BMC Public Health 2007 7 200 207 10.1186/1471-2458-7-200 17688689 PMC1988820

[B16] Semenza JC McCullough JE Flanders WD McGeehin MA Lumpkin JR Excess hospital admissions during the July 1995 heat wave in Chicago Am J Prev Med 1999 16 269 277 10.1016/S0749-3797(99)00025-2 10493281

[B17] Nitschke M Tucker GR Hansen AL Williams S Zhang Y Bi P Impact of two recent extreme heat episodes on morbidity and mortality in Adelaide South Australia: a case-series analysis Environ Health 2011 10 42 10.1186/1476-069X-10-42 21592410 PMC3116460

[B18] Kovats RS Hajat S Wilkinson P Contrasting patterns of mortality and hospital admissions during hot weather and heat waves in Greater London, UK Occup Environ Med 2004 61 893 898 10.1136/oem.2003.012047 15477282 PMC1757853

[B19] Cerutti B Tereanu C Domenighetti G Cantoni E Gaia M Bolgiani I Lazzaro M Cassis I Temperature related mortality and ambulance service interventions during the heat waves of 2003 in Ticino (Switzerland) Soz Praventivmed 2006 51 185 193 10.1007/s00038-006-0026-z 17193780

[B20] Knowlton K Rotkin-Ellman M King G Margolis HG Smith D Solomon G Trent R English P The 2006 California heat wave: impacts on hospitalizations and emergency department visits Environ Health Perspect 2009 117 61 67 19165388 10.1289/ehp.11594 PMC2627866

[B21] Dhainaut JF Claessens YE Ginsburg C Riou B Unprecedented heat-related deaths during the 2003 heat wave in Paris: consequences on emergency departments Crit Care 2004 8 1 2 10.1186/cc2404 14975035 PMC420061

[B22] Astrom DO Forsberg B Rocklov J Heat wave impact on morbidity and mortality in the elderly population: a review of recent studies Maturitas 2011 69 99 105 10.1016/j.maturitas.2011.03.008 21477954

[B23] Kenney WL Munce TA Invited review: aging and human temperature regulation J Appl Physiol 2003 95 2598 2603 14600165 10.1152/japplphysiol.00202.2003

[B24] Bouchama A Dehbi M Mohamed G Matthies F Shoukri M Menne B Prognostic factors in heat wave related deaths: a meta-analysis Arch Intern Med 2007 167 2170 2176 10.1001/archinte.167.20.ira70009 17698676

[B25] Conti S Masocco M Meli P Minelli G Palummeri E Solimini R Toccaceli V Vichi M General and specific mortality among the elderly during the 2003 heat wave in Genoa (Italy) Environ Res 2007 103 267 274 10.1016/j.envres.2006.06.003 16890219

[B26] Hansen AL Bi P Ryan P Nitschke M Pisaniello D Tucker G The effect of heat waves on hospital admissions for renal disease in a temperate city of Australia Int J Epidemiol 2008 37 1359 1365 10.1093/ije/dyn165 18710886

[B27] Hansen A Bi P Nitschke M Ryan P Pisaniello D Tucker G The effect of heat waves on mental health in a temperate Australian city Environ Health Perspect 2008 116 1369 1375 10.1289/ehp.11339 18941580 PMC2569097

[B28] Kaiser R Rubin CH Henderson AK Wolfe MI Kieszak S Parrott CL Adcock M Heat-related death and mental illness during the 1999 Cincinnati heat wave Am J Forensic Med Pathol 2001 22 303 307 10.1097/00000433-200109000-00022 11563746

[B29] Bi P Williams S Loughnan M Lloyd G Hansen A Kjellstrom T Dear K Saniotis A The effects of extreme heat on human mortality and morbidity in Australia: implications for public health Asia Pac J Public Health 2011 23 27S 36S 10.1177/1010539510391644 21247972

[B30] Tong S Ren C Becker N Excess deaths during the 2004 heatwave in Brisbane, Australia Int J Biometeorol 2010 54 393 400 10.1007/s00484-009-0290-8 20049484

[B31] Australian Bureau of Statistics (ABS) Population by Age and Sex, Regions of Australia, 2009. http://www.abs.gov.au/ausstats/abs@.nsf/Products/3235.0~2009~Main+Features~New+South+Wales

[B32] Bureau of Meteorology (BOM) An exceptional summer heatwave in greater Sydney and the Hunter Valley. Special Climate Statement 27 http://www.bom.gov.au/climate/current/statements/scs27.pdf

[B33] Heffernan R Mostashari F Das D Karpati A Kulldorff M Weiss D Syndromic surveillance in public health practice, New York City Emerg Infect Dis 2004 10 858 864 15200820 10.3201/eid1005.030646

[B34] Marx MA Rodriguez CV Greenko J Das D Heffernan R Karpati AM Mostashari F Balter S Layton M Weiss D Diarrheal illness detected through syndromic surveillance after a massive power outage: New York City, August 2003 Am J Public Health 2006 96 547 553 10.2105/AJPH.2004.061358 16380562 PMC1470517

[B35] Yuan CM Love S Wilson M Syndromic surveillance at hospital emergency departments-southeastern Virginia MMWR Morb Mortal Wkly Rep 2004 53 Suppl 56 58 15714630

[B36] Josseran L Fouillet A Caillere N Brun-Ney D Ilef D Brucker G Medeiros H Astagneau P Assessment of a syndromic surveillance system based on morbidity data: results from the Oscour network during a heat wave PLoS One 2010 5 e11984 e11991 10.1371/journal.pone.0011984 20711252 PMC2918496

[B37] Leonardi GS Hajat S Kovats RS Smith GE Cooper D Gerard E Syndromic surveillance use to detect the early effects of heat-waves: an analysis of NHS direct data in England Soz Praventivmed 2006 51 194 201 10.1007/s00038-006-5039-0 17193781

[B38] Muscatello DJ Churches T Kaldor J Zheng W Chiu C Correll P Jorm L An automated, broad-based, near real-time public health surveillance system using presentations to hospital Emergency Departments in New South Wales, Australia BMC Public Health 2005 5 141 152 10.1186/1471-2458-5-141 16372902 PMC1361771

[B39] Anderson BG Bell ML Weather-related mortality: how heat, cold, and heat waves affect mortality in the United States Epidemiology 2009 20 205 213 10.1097/EDE.0b013e318190ee08 19194300 PMC3366558

[B40] Filleul L Cassadou S Medina S Fabres P Lefranc A Eilstein D Le Tertre A Pascal L Chardon B Blanchard M The relation between temperature, ozone, and mortality in nine French cities during the heat wave of 2003 Environ Health Perspect 2006 114 1344 1347 10.1289/ehp.8328 16966086 PMC1570046

[B41] Hu W Mengersen K McMichael A Tong S Temperature, air pollution and total mortality during summers in Sydney, 1994-2004 Int J Biometeorol 2008 52 689 696 10.1007/s00484-008-0161-8 18506490

[B42] Lin S Luo M Walker RJ Liu X Hwang SA Chinery R Extreme high temperatures and hospital admissions for respiratory and cardiovascular diseases Epidemiology 2009 20 738 746 10.1097/EDE.0b013e3181ad5522 19593155

[B43] New South Wales Goverment Office of Environment and Heritage Air quality data http://www.environment.nsw.gov.au/AQMS/index.htm

