# Supplementary material for: Emergency department visits, ambulance calls, and mortality associated with an exceptional heat wave in Sydney, Australia, 2011: a time-series analysis
Source: Environ Health. 2012 Jan 24;11:3. doi: 10.1186/1476-069X-11-3 (PMC3292446; doi:10.1186/1476-069X-11-3)
Supplement: Additional file 1 — Table S1. List of SNOMED codes used to identify ED visits for dehydration and heat-related syndromes. [file 1476-069X-11-3-S1.PDF]

Table S1: List of SNOMED codes used to identify ED visits for dehydration and heat-related syndromes

Syndrome	Concept ID	Concept name
Dehydration	162685008	On examination - dehydrated (disorder)
	182783002	Dehydration monitoring (regime/therapy)
	190894003	Isonatremic dehydration (disorder)
	190896001	Dehydration NEC (disorder)
	190897005	Volume depletion NOS (disorder)
	234178003	Absolute hypovolemia (disorder)
	234179006	Relative hypovolemia (disorder)
	2520005	AIDS with volume depletion (disorder)
	28560003	Hypovolemia (disorder)
	34095006	Dehydration (disorder)
	44759006	Volume depletion, extrarenal loss (disorder)
	47417003	Volume depletion, gastrointestinal loss (disorder)
	54480007	Volume depletion, renal, due to output loss (renal deficit, disorder) (disorder)
	87571007	Volume depletion, renal, due to effector loss (hormonal deficit, disorder) (disorder)
Heat stroke, sunstroke, heat syncope, heat exhaustion and other heat effects	12979003	Transient heat fatigue (disorder)
	16209006	Anhidrotic heat exhaustion (disorder)
	18615009	Sunstroke (disorder)
	212937007	Heat stroke and sunstroke (disorder)
	212938002	(Siriasis) or (thermoplegia) or (heat stroke, unspecified)
	212939005	Heat stroke or sunstroke NOS (disorder)
	212941006	Heat: [exhaustion, unspecified] or [prostration NOS]
	212942004	Other heat effects (disorder)
	212943009	Heat effects NOS (disorder)

Table S1: List of SNOMED codes used to identify ED visits for dehydration and heat-related syndromes (continued)

Syndrome	Concept ID	Concept name
Heat stroke, sunstroke, heat syncope, heat exhaustion and other heat effects (continued)	213711005	[X]Other heat effects (disorder)
	221628008	[X]Exposure to excessive natural heat (event)
	221629000	[X]Exposure to excessive natural heat, occurrence at home (event)
	221630005	[X]Exposure to excessive natural heat, occurrence in residential institution (event)
	221631009	[X]Exposure to excessive natural heat, occurrence at school, other institution and public administrative area (event)
	221632002	[X]Exposure to excessive natural heat, occurrence at sports and athletics area (event)
	221633007	[X]Exposure to excessive natural heat, occurrence on street and highway (event)
	221634001	[X]Exposure to excessive natural heat, occurrence at trade and service area (event)
	221635000	[X]Exposure to excessive natural heat, occurrence at industrial and construction area (event)
	221636004	[X]Exposure to excessive natural heat, occurrence on farm (event)
	221637008	[X]Exposure to excessive natural heat, occurrence at other specified place (event)
	221638003	[X]Exposure to excessive natural heat, occurrence at unspecified place (event)
	241971009	Heat prostration NOS (disorder)
	269276002	Heat stroke, unspecified (disorder)
	269277006	Heat exhaustion
	52072009	Heat stroke (disorder)
	55017000	Heat edema (disorder)
	76149006	Heat exhaustion due to salt depletion (disorder)
	87108006	Heat cramps (disorder)
	8824003	Effects of heat AND/OR light (disorder)
	89797005	Heat syncope (disorder)
	95868006	Heat exhaustion (disorder)
